# Gallic Acid Attenuates Angiotensin II-Induced Hypertension and Vascular Dysfunction by Inhibiting the Degradation of Endothelial Nitric Oxide Synthase

**DOI:** 10.3389/fphar.2020.01121

**Published:** 2020-07-22

**Authors:** Xiao Yan, Qi-Yu Zhang, Yun-Long Zhang, Xiao Han, Shu-Bin Guo, Hui-Hua Li

**Affiliations:** ^1^ Emergency Medicine Clinical Research Center, Beijing Chao-Yang Hospital, Capital Medical University, and Beijing Key Laboratory of Cardiopulmonary Cerebral Resuscitation, Beijing, China; ^2^ Department of Cardiology, Institute of Cardiovascular Diseases, First Affiliated Hospital of Dalian Medical University, Dalian, China

**Keywords:** gallic acid, angiotensin II, hypertension, immunoproteasome, eNOS degradation

## Abstract

Hypertension is a major cause of heart attack and stroke. Our recent study revealed that gallic acid (GA) exerts protective effects on pressure overload-induced cardiac hypertrophy and dysfunction. However, the role of GA in angiotensin II (Ang II)-induced hypertension and vascular remodeling remains unknown. C57BL/6J mice were subjected to saline and Ang II infusion. Systolic blood pressure was measured using a tail-cuff system. Vascular remodeling and oxidative stress were examined by histopathological staining. Vasodilatory function was evaluated in the aortic ring. Our findings revealed that GA administration significantly ameliorated Ang II-induced hypertension, vascular inflammation, and fibrosis. GA also abolished vascular endothelial dysfunction and oxidative stress in Ang II-infused aortas. Mechanistically, GA treatment attenuated Ang II-induced upregulation of the immunoproteasome catalytic subunits β2i and β5i leading to reduction of the trypsin-like and chymotrypsin-like activity of the proteasome, which suppressed degradation of endothelial nitric oxide synthase (eNOS) and reduction of nitric oxide (NO) levels. Furthermore, blocking eNOS activity by using a specific inhibitor (_L_-*N* ^G^-nitroarginine methyl ester) markedly abolished the GA-mediated beneficial effect. This study identifies GA as a novel immunoproteasome inhibitor that may be a potential therapeutic agent for hypertension and vascular dysfunction.

## Introduction

Hypertension remains a major risk factor for cardiovascular events, chronic kidney disease, and heart failure (Carey et al., 2018). Recent studies have revealed that vascular inflammation and oxidative stress, which are hallmarks of endothelial dysfunction, contribute to the pathogenesis of hypertension (Wang et al., 2016; Konukoglu and Uzun, 2017; Lang et al., 2019). Importantly, endothelial nitric oxide synthase (eNOS) acts as a key regulator of vasodilation and vasoprotection in physiological and pathological states, respectively (Garcia and Sessa, 2019). eNOS-derived nitric oxide (NO) inhibits platelet aggregation and adhesion, vascular smooth muscle proliferation, and vascular inflammation (Forstermann and Sessa, 2012). Increasing evidence suggests that sustained hypertensive stimuli such as reactive oxygen species and angiotensin II (Ang II), suppress eNOS expression and NO bioavailability, thereby leading to a reduction of endothelium-dependent vasodilation in the vasculature (Gryglewski et al., 1986; Schrader et al., 2007). Inhibition of basal eNOS activity by administration of _L_-*N* ^G^-nitroarginine methyl ester (L-NAME) increases vasoconstriction, pathological vascular remodeling, and blood pressure (Ribeiro et al., 1992). In contrast, animal and pre-clinical studies have demonstrated that gene delivery of eNOS is effective in inhibiting vascular injury and promoting endothelial regeneration (Cooney et al., 2007). It is interesting to note that the ubiquitin-proteasome system (UPS) is involved in the regulation of eNOS activity (Stangl et al., 2004). However, the underlying mechanisms by which the proteasome modulates eNOS stability in Ang II-induced hypertension and vascular dysfunction remain unclear.

Natural compounds have been shown to reduce the risk factors of cardiovascular diseases (Pandey and Rizvi, 2009). As a plant-derived phenolic acid, gallic acid (GA) has been shown to exert beneficial effects on myocardial hypertrophy, fibrosis, and oxidative stress in response to various hypertrophic stimuli (Ryu et al., 2016; Yan et al., 2019). We have recently found that GA administration attenuates pressure overload-induced cardiac hypertrophic remodeling by promoting the autophagy-dependent degradation of epidermal growth factor receptor, glycoprotein 130, and calcineurin A (Yan et al., 2019). Moreover, several studies have revealed that GA inhibits hypertension in spontaneously hypertensive rats (SHRs) and L-NAME-treated mice (Kang et al., 2015; Jin et al., 2017a). Intriguingly, an *in vitro* finding demonstrates that GA improves endothelial injury by suppressing the chymotrypsin-like activity of the proteasome (Kam et al., 2014). However, there is little information about the role of GA in the regulation of endothelial dysfunction and hypertension in Ang II-infused mice.

Here, we provide novel evidence that GA administration significantly attenuated Ang II-induced hypertension and vascular remodeling, which was associated with an improvement of endothelium-dependent vascular dysfunction. Furthermore, GA markedly blocked the activity and expression of the immunoproteasome catalytic subunits β2i and β5i, leading to the suppression of eNOS degradation and the reduction of NO levels in Ang II-infused mice. Collectively, these data indicate that GA ameliorates vascular injury likely by inhibiting immunoproteasome-dependent eNOS degradation, and may serve as a promising candidate for treating hypertension.

## Materials and Methods

### Animal Models and Experimental Protocols

Wild-type (WT) C57BL/6 mice (male, 8–12 weeks) were purchased from Jackson Laboratory (Bar Harbor, ME, USA). The procedures were approved by the Animal Care and Use Committee of Capital Medical University (AEE1-2016-045). All investigations were conformed to the Guide for the Care and Use of Laboratory Animals published by the U.S. National Institutes of Health (NIH Publication No.85-23, revised 1996). The Ang II-induced hypertension model was performed by 14-day subcutaneous infusion of Ang II (490 ng/kg/min; Sigma-Aldrich, St. Louis, MO) or saline using osmotic mini-pumps (Alzet MODEL 1007D; DURECT, Cupertino, CA) as previously described (Wang et al., 2016; Lang et al., 2019). The systolic blood pressure (SBP) and heart rate (HR) of mice was gauged by a tail-cuff system (SoftronBP-98A; Softron, Tokyo, Japan).

Mice were orally gavaged with vehicle or GA (Sigma-Aldrich) at doses of 5 or 20 mg/kg body weight (BW) daily and randomly subjected to the saline or Ang II treatment. A specific eNOS inhibitor L-NAME (Sigma-Aldrich) was administrated in the drinking water (1 mg/ml) (Boe et al., 2013). After 2 weeks of Ang II or saline infusion, animals were anaesthetized by intraperitoneal injection of an overdose of pentobarbital (100 mg/kg, Sigma-Aldrich). The aortas were harvested and prepared for further histological and molecular experiments.

### Vascular Relaxation Analysis

The thoracic aortas were isolated and cut into 4-mm segments and gently mounted on force transducers (Power Laboratory, AD Instruments, Bella Vista, Australia) in organ chambers. The samples were challenged with 60 mmol/L KCl, and then stimulated by noradrenaline. The vascular responses to increasing concentrations of acetylcholine (ACh) and sodium nitroprusside (SNP) were detected as described previously (Wang et al., 2016; Lang et al., 2019).

### Histopathology

The aortic tissues were fixed in 4% paraformaldehyde and embedded in paraffin. Sections (5 μm) were stained with haematoxylin and eosin (H&E) and Masson’s trichrome reagent, as well as dihydroethidine (DHE, 1 μM in PBS; Sigma-Aldrich) in accordance with standard procedures (Wang et al., 2016; Lang et al., 2019). Immunohistochemistry staining was performed with the anti-Mac-2 antibody (1:200 dilution; Santa Cruz Biotechnology Inc., Dallas, TX). Images were detected by Nikon Labophot 2 microscope (Nikon, Tokyo, Japan) and analyzed using Image J software (US National Institutes of Health, Bethesda, MD).

### Proteasome Activity

The aortic proteasome activity was measured using fluorogenic peptide substrates as previously described (Li et al., 2015; Chen et al., 2019; Li J. et al., 2019). In brief, the protein of aorta was isolated with HEPES buffer (50 mM, pH 7.5) consist of 20 mmol/L KCl, 5 mmol/L MgCl_2_, and 1 mmol/L dithiothreitol. Z-LLE-AMC (45 μmol/L), Ac-RLR-AMC (40 μmol/L), and Suc-LLVY-AMC (18 μmol/L) were utilized to evaluate the caspase-like, trypsin-like, and chymotrypsin-like activity, respectively. Twenty micrograms of protein were added to 100 μl of the HEPES buffer containing the fluorogenic peptide substrates and incubated for 10 min at 37°C. The fluorescence intensity was gauged with the excitation at 380 nm and emission at 460 nm.

### Quantitative Real-Time PCR Analysis

Total RNA was extracted from aorta tissues by Trizol Reagent (Invitrogen, Carlsbad, CA) and reverse-transcribed according to the manufacturer’s protocol (Wang et al., 2016; Lang et al., 2019). PCR amplification was performed using 1–2 μg of cDNA and gene-specific primers (Sangon Biotech, Shanghai, China), which are listed in **Supplementary Table 1**. Quantitative real-time PCR (qPCR) was performed with an iCycler IQ system (Bio-Rad, CA), and the transcript quantities were normalized to the amount of glyceraldehyde-3-phosphate dehydrogenase (GAPDH).

### Western Blot Analysis

Total proteins were isolated from snap-frozen aorta samples using RIPA buffer containing protease inhibitors (Solarbio Science Technology Co, China). The lysates (40–50 μg) were separated by electrophoresis in 8–12% SDS-PAGE gels, transferred to the polyvinylidene difluoride (PVDF) membranes (Bio-Rad), and incubated with the primary antibodies against β2i (Abcam, London, UK), β5i (Abcam), p-eNOS^1177^ (Cell Signaling Technologies, Boston, MA), eNOS (Cell Signaling Technologies), and GAPDH (Proteintech Group Inc, Rosemont, IL). The horseradish peroxidase-conjugated anti-mouse or anti-rabbit IgG were purchased from Cell Signaling Technologies. All blots were analyzed by the Image J software and normalized to GAPDH.

### NO Assay

The aortic and serum NO levels were evaluated using a colorimetric assay kit (Nanjing Jiancheng Biological Company, China) in accordance with the manufacturer’s protocol.

### Statistical Analysis

All results are presented as mean ± standard error of the mean (SEM). The normality test (Shapiro-Wilk) was used to determine whether the data were normally distributed. The student t test was used to compare the significant difference between two groups in normal distribution. If the data were not normally distributed, the Mann-Whitney test was performed. One-way ANOVA following Newman-Keuls multiple comparison test was performed to evaluate the significance of difference between the means of groups. For blood pressure data and ACh- or SNP-induced vasodilation tests in aortic rings, repeated-measures ANOVA analysis of variance was utilized. If the ANOVA analysis demonstrated a significant effect, *post hoc* comparisons were made pairwise with the Fisher least significant difference test. *P* < 0.05 was considered statistically significant.

## Results

### GA Reduces Ang II-Induced Hypertension, Vascular Remodeling, and Inflammation

**Figure 1:**
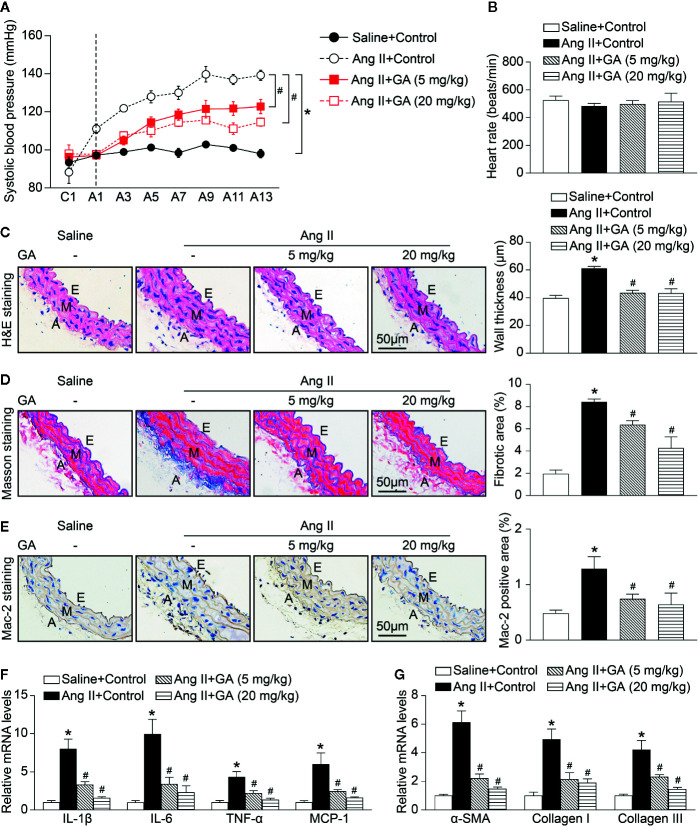
Gallic acid (GA) ameliorates hypertension, vascular inflammation, and fibrosis in Ang II-infused mice. WT mice were orally gavaged with vehicle or GA (5 or 20 mg/kg BW) for 14 days in the presence of saline or Ang II infusion (490 ng·kg^-1^min^-1^). **(A)** Average systolic blood pressure of vehicle or GA-treated mice before and after Ang II treatment obtained by telemetry (n=6). **(B)** Heart rate was assessed by the noninvasive tail-cuff method in vehicle- and GA-treated mice after saline and Ang II infusion (n=6). **(C)** Representative images of haematoxylin and eosin (H&E) staining of the thoracic aorta (left), and quantification of the wall thickness of each group (right, n=6). **(D)** Representative images of Masson’s trichrome staining of the thoracic aorta (left), and quantification of the percentage of fibrotic area (right, n=6). **(E)** Representative images of immunohistochemical staining of aorta sections with anti-Mac-2 antibody (left), and quantification of Mac-2-positive macrophages (right, n=6). A indicates adventitia; E, endothelium; M, media. Scale bar: 50 μm. **(F)** Quantitative real-time PCR (qPCR) analyses of the mRNA expression of IL-1β, IL-6, TNF-α, and MCP-1 in the aorta (n=6). **(G)** qPCR analyses of α-SMA, collagen I, and collagen III mRNA expression levels (n=6). GAPDH as the internal control. For blood pressure data, repeated-measures ANOVA was used. If the ANOVA analysis demonstrated a significant effect, *post hoc* comparisons were made pairwise with the Fisher least significant difference test. One-way ANOVA following Newman-Keuls multiple comparison test was utilized to evaluate the significance of difference between the means of groups. *P < 0.05 versus saline + control, ^#^ *P* < 0.05 versus Ang II + control.

To investigated the functional role of GA in the regulation of blood pressure in Ang II-infused mice, wild-type (WT) mice were treated with different doses of GA (5 or 20 mg/kg BW) and infused with Ang II (490 ng/kg/min). Systolic blood pressure (SBP) was measured by the noninvasive tail-cuff method. We found that Ang II infusion for 2 weeks significantly increased SBP compared with saline-treated controls, whereas this increase was markedly reduced by GA (5 or 20 mg/kg BW) in Ang II-treated mice (**Figure 1A**). The heart rate was not significantly altered in the vehicle- and GA-treated mice after saline or Ang II infusion (**Figure 1B**). Moreover, Ang II-induced increases in aortic wall thickening, collagen deposition, and the accumulation of Mac-2-positive macrophages were also blunted in GA-treated mice (**Figures 1C–E**). Accordingly, Ang II-induced upregulation of the mRNA expression of proinflammatory and fibrotic genes (interleukin [IL]-1β, IL-6, tumor necrosis factor [TNF]-α, monocyte chemoattractant protein-1, α-smooth muscle actin, collagen I, and collagen III) in Ang II-infused aortas was remarkably attenuated in GA-treated mice in a dose-dependent manner (**Figures 1F, G**). These results indicate that the administration of GA improves Ang II-induced hypertension and vascular injury.

### GA Blocks Vascular Dysfunction and Oxidative Stress in Ang II-Infused Mice

**Figure 2:**
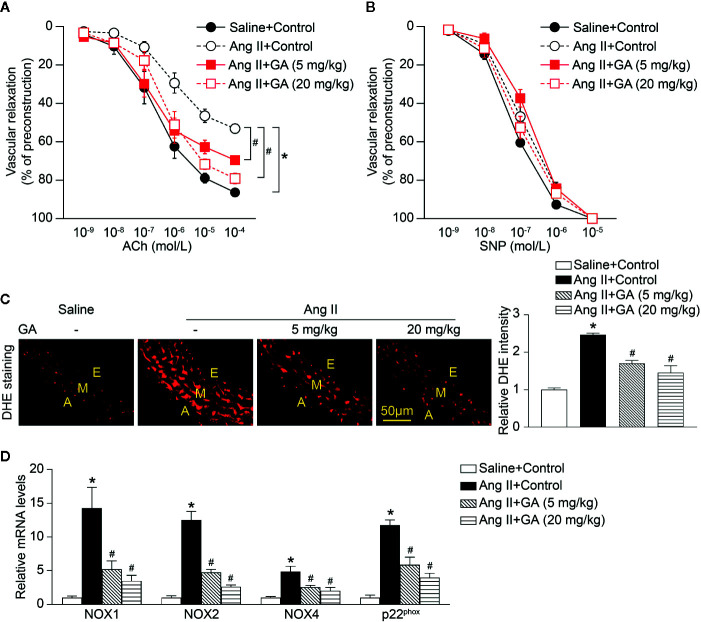
Gallic acid (GA) attenuates Ang II-induced vascular dysfunction and oxidative stress. **(A)** Dose-response curves of endothelium-dependent relaxation (ACh, n=6). **(B)** Dose-response curves of endothelium-independent relaxation in response to SNP (n=6). **(C)** Representative images of DHE staining of the aortic superoxide production (left), and quantiﬁcation of fluorescence intensity (right, n=6). A indicates adventitia; E, endothelium; M, media. Scale bar: 50 μm. **(D)** Quantitative real-time PCR (qPCR) analyses of the mRNA expression levels of NOX1, NOX2, NOX4, and p22^phox^ (n=4). Glyceraldehyde-3-phosphate dehydrogenase (GAPDH) as an internal control. For ACh- or sodium nitroprusside (SNP)-induced vasodilation tests in aortic rings, repeated-measures ANOVA analysis of variance was utilized. If the analysis of variance demonstrated a significant effect, *post hoc* comparisons were made pairwise with the Fisher least significant difference test. One-way ANOVA following Newman-Keuls multiple comparison test was used to evaluate the significance of difference between the means of groups. *P < 0.05 versus saline + control, ^#^ *P* < 0.05 versus Ang II + control.

To determine whether GA treatment suppressed vascular dysfunction, we evaluated *ex vivo* vascular function in vehicle- or GA-treated mice in response to Ang II. Two-week Ang II infusion significantly impaired endothelium-dependent vasodilatation to acetylcholine (ACh) compared with saline control (**Figure 2A**). However, this effect was dose-dependently improved in GA-treated mice (**Figure 2A**). Consistent with previous findings (Wang et al., 2016), there was no statistically significant difference in endothelium-independent vasodilatation to sodium nitroprusside (SNP) between Ang II- and saline-treated mice (**Figure 2B**). Moreover, SNP-induced vasodilatation was not changed in Ang II-infused mice after GA administration (**Figure 2B**). These data suggest that GA prevented endothelial dysfunction in Ang II-infused mice.

Recent studies have found that vascular superoxide production contributes to endothelial dysfunction in the Ang II-treated mouse model (Rajagopalan et al., 1996; Wang et al., 2016). As indicated in **Figure 2C**, Ang II infusion for 2 weeks markedly increased the formation of aortic superoxide as characterized by dihydroethidium (DHE) staining, whereas this effect was abolished in GA-treated mice in a dose-dependent manner. Furthermore, the upregulated mRNA levels of the NADPH oxidase catalytic subunits NOX1, NOX2, and NOX4, and p22^phox^ in Ang II-infused aortas were significantly ameliorated in mice treated with GA (**Figure 2D**). Therefore, these findings illustrate that the GA treatment attenuates Ang II-induced aortic superoxide formation, which is associated with vascular dysfunction.

### GA Inhibits eNOS Degradation by Attenuating Immunoproteasome Activity in Ang II-Treated Mice

**Figure 3:**
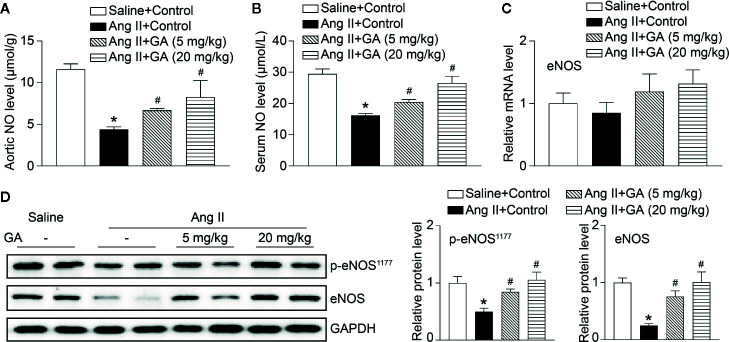
Gallic acid (GA) downregulates the degradation of endothelial nitric oxide synthase (eNOS) and the reduction of nitric oxide (NO) levels in Ang II-treated aortas. **(A)** Measurement of NO levels in the aorta by the colorimetric assay (n=6). **(B)** NO levels in the serum (n=6). **(C)** The qPCR analysis of eNOS mRNA expression in the aorta (n=6). **(D)** Representative immunoblotting analyses of the protein expression of p-eNOS^1177^ and eNOS (left), and quantification of the relative protein levels (right, n=4). Glyceraldehyde-3-phosphate dehydrogenase (GAPDH) as an internal control. One-way ANOVA following Newman-Keuls multiple comparison test was used to evaluate the significance of difference between the means of groups. *P < 0.05 versus saline + control, ^#^ *P* < 0.05 versus Ang II + control.

It is well established that eNOS-derived NO exerts essential effects on vascular dilation (Forstermann and Sessa, 2012). We revealed that Ang II infusion for 2 weeks significantly reduced aortic and serum NO levels, and this effect was diminished after GA treatment (**Figures 3A, B**). However, the mRNA level of eNOS, inducible NO synthase (iNOS), Ang II type I receptor (AT1R), and AT2R were not altered in Ang II-infused mice after GA treatment (**Figure 3C** and **Supplementary Figure 1**). It is interesting to note that GA treatment reversed the Ang II-induced downregulation of p-eNOS^1177^ and eNOS protein expression in the aortas (**Figure 3D**) and human umbilical vein endothelial cells (HUVECs) (**Supplementary Figure 2**), suggesting that reduction of eNOS expression occurs at protein level.

**Figure 4:**
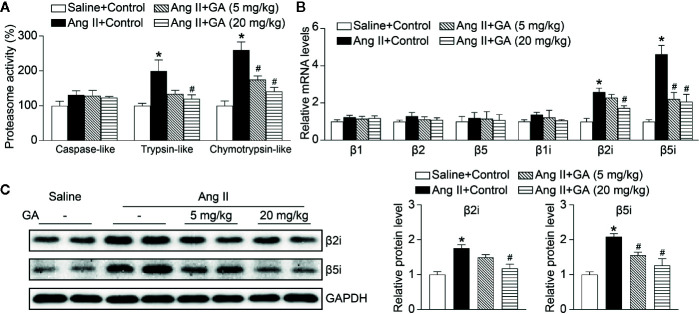
Gallic acid (GA) abolishes immunoproteasome activity and catalytic subunit expression in the aorta after Ang II infusion. **(A)** The caspase-like, trypsin-like, and chymotrypsin-like activity of the proteasome in aortas in response to Ang II infusion with vehicle or GA treatment (n=4). **(B)** Quantitative real-time PCR (qPCR) analyses of the mRNA expression of β1, β2, β5, β1i, β2i, and β5i. **(C)** Representative immunoblotting analyses of the protein expression of β2i and β5i (left), and quantification of the relative protein levels (right, n=4). Glyceraldehyde-3-phosphate dehydrogenase (GAPDH) as an internal control. One-way ANOVA following Newman-Keuls multiple comparison test was used to evaluate the significance of difference between the means of groups. *P < 0.05 versus saline + control, ^#^ *P* < 0.05 versus Ang II + control.

Since the proteasome-mediated regulation of eNOS stability contributes to endothelial function and vasodilation in the aorta (Stangl et al., 2004), we then investigated whether GA affects proteasome activity and expression of catalytic subunits. As expected, Ang II infusion significantly induced increase of the trypsin-like and chymotrypsin-like activity of the proteasome as well as the mRNA levels of the immunoproteasome subunits β2i and β5i, but did not influence other standard and catalytic subunits (β1, β2, β5, and β1i) in the aorta, and the increase was dose-dependently abolished by GA (**Figures 4A, B**). Moreover, GA treatment also markedly reduced the protein levels of β2i and β5i in Ang II-treated aortas and HUVECs (**Figure 4C** and **Supplementary Figure 2**). Overall, these results indicate that GA blunts the Ang II-induced reduction of NO and degradation of eNOS likely by suppressing the activity and expression of β2i and β5i in the aorta.

### Blockage of eNOS Activity Diminishes GA-Mediated Protective Effects on Hypertension in Ang II-Treated Mice

**Figure 5:**
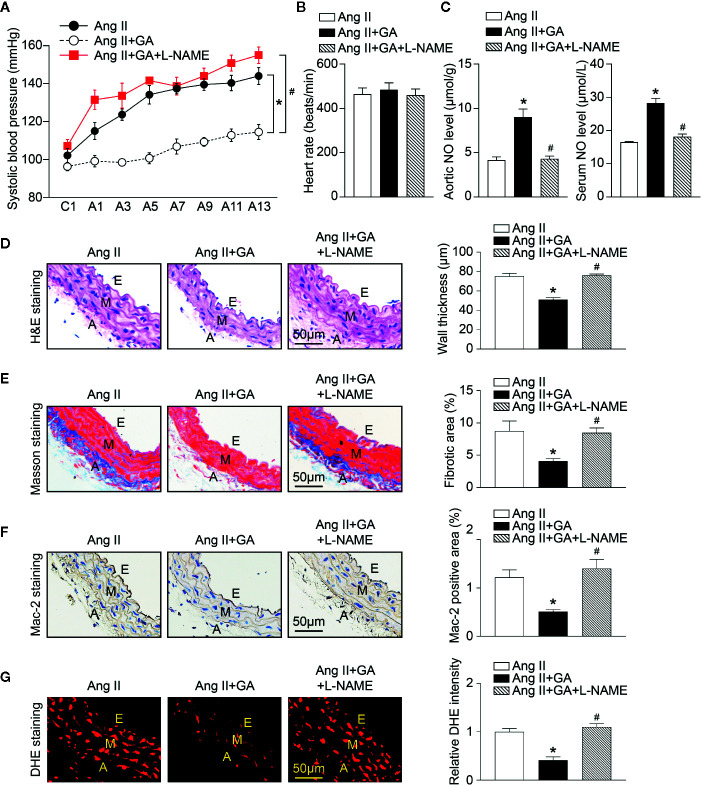
Blocking endothelial nitric oxide synthase (eNOS) activity inhibits gallic acid (GA)-mediated protective effect on Ang II-induced hypertension and vascular remodeling. **(A)** Average systolic blood pressure was gauged by the noninvasive tail-cuff method in vehicle or GA-treated mice before and after Ang II infusion together with _L_-*N* ^G^-nitroarginine methyl ester (L-NAME) treatment (1 mg/ml drinking water, n=6). **(B)** Heart rate of vehicle or GA-treated mice after Ang II infusion together with L-NAME treatment (n=6). **(C)** Nitric oxide (NO) levels in the aorta (left, n=6) and serum (right, n=6). **(D)** Haematoxylin and eosin (H&E) staining in the aorta (left). Quantification of aortic wall thickness (right, n=6). **(E)** Masson’s trichrome staining for aortic fibrosis (left). Quantification of the fibrotic area (right, n=6). **(F)** Immunohistochemical staining of aorta sections with anti-Mac-2 antibody (left), and quantification of Mac-2-positive macrophages (right, n=6). **(G)** Dihydroethidine (DHE) staining of superoxide production in the aorta (left). Quantiﬁcation of DHE fluorescence intensity (right, n=6). A indicates adventitia; E, endothelium; M, media. Scale bar: 50 μm. For blood pressure data, repeated-measures ANOVA was used. If the ANOVA analysis demonstrated a significant effect, *post hoc* comparisons were made pairwise with the Fisher least significant difference test. After the normality test (Shapiro-Wilk), the student t test was performed to compare the significant difference between two groups in normal distribution, and the Mann-Whitney test was used for the data that were not normally distributed. *P < 0.05 versus Ang II, ^#^ *P* < 0.05 versus Ang II + GA.

To test whether eNOS is involved in Ang II-induced hypertension and vascular dysfunction after GA administration in mice, we treated wild-type mice with GA in the presence or absence of a special eNOS inhibitor (L-NAME) for 2 weeks. In agreement with our previous results (**Figures 1** and **2**), the administration of GA abolished the Ang II-induced increase of SBP and decrease of aortic and serum NO levels (**Figures 5A, C**). The heart rate was not changed in vehicle- or GA-treated mice after Ang II infusion (**Figure 5B**). Ang II-induced increases in aortic thickening, collagen deposition, the accumulation of Mac-2-positive macrophages, and superoxide formation were also ameliorated in GA-treated mice (**Figures 5D–G**). However, these effects were reversed by L-NAME treatment (**Figures 5A, C–F**). Accordingly, L-NAME did not affect the heart rate in Ang II-infused mice (**Figure 5B**). Collectively, these results suggest that GA suppresses hypertension and vascular injury by attenuating the degradation of eNOS after Ang II infusion.

## Discussion

**Figure 6:**
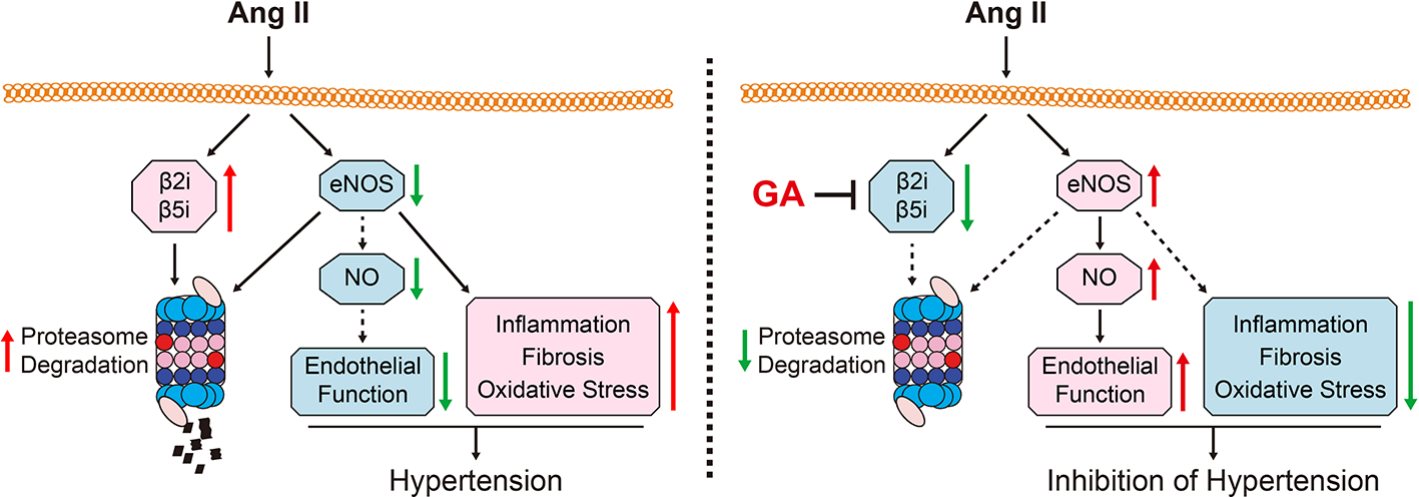
A working diagram of gallic acid (GA)-mediated beneficial effect on the Ang II-infused mouse model. Ang II infusion increases the activity and the expression of the immunoproteasome subunits β2i and β5i, which induces the degradation of endothelial nitric oxide synthase (eNOS) and the reduction of nitric oxide (NO) levels thereby leading to hypertension and impairment of vascular function. Conversely, GA administration attenuates these effects. Ang II, angiotensin II; eNOS, endothelial nitric oxide synthase; GA, gallic acid; NO, nitric oxide.

In this study, we demonstrated that GA administration significantly ameliorated the Ang II-induced development of hypertension and vascular remodeling in mice. Mechanistically, GA reduced the activity and expression of the immunoproteasome catalytic subunits β2i and β5i, which abolished the degradation of eNOS, leading to the production of NO and improvement of endothelium-dependent vascular dysfunction (**Figure 6**). Therefore, our study provides evidence that GA represents a novel immunoproteasome inhibitor and may be a potential therapeutic agent for hypertension and vascular dysfunction.

The renin-angiotensin-aldosterone system (RAAS) plays a critical role in the initiation and development of hypertension. As the most powerful vasoconstrictor in the RAAS, Ang II is involved in the regulation of multiple processes, including inflammation, fibrosis, and oxidative stress (Forrester et al., 2018). Current strategies for treating hypertension comprise adrenoceptor antagonists, angiotensin-converting enzyme inhibitors (ACEIs), angiotensin receptor blockers, and mineralocorticoid antagonists; however, their efficacy remains low (Cai and Calhoun, 2017). Thus, there is an urgent need to identify candidate therapeutic approaches for treating hypertensive diseases. GA is a food-derived polyphenol compound that plays beneficial roles in improving hypertension, vascular dysfunction, and cardiac hypertrophic remodeling in several hypertensive models (Jin et al., 2017a; Jin et al., 2017b). In Ang II-treated H9c2 cells and SHRs, GA attenuates GATA4-induced NOX activity, which reduces oxidative stress and blood pressure (Jin et al., 2017b). Moreover, GA ameliorates L-NAME-induced hypertension and myocardial fibrosis by modulating histone deacetylase 1 and 2 (Jin et al., 2017a). In this study, our data revealed that GA abolished Ang II-induced hypertension and vascular dysfunction, which was associated with the suppression of the activity and expression of the immunoproteasome subunits β2i and β5i, leading to decreased eNOS degradation.

The 26S proteasome accounts for the majority of protein degradation in mammalian cells (Angeles et al., 2012; Thibaudeau and Smith, 2019). As the core part of the UPS, the 26S proteasome is composed of the 20S core protease and 19S regulatory particle. The 20S proteasome contains two pairs of inner β-rings and three catalytic β-subunits including β1 (PMSB6), β2 (PMSB7), and β5 (PMSB5), which exhibit caspase-like, trypsin-like, and chymotrypsin-like activity, respectively. After stimulation with interferon-γ or TNF-α, the standard β-subunits of the constitutive proteasome are replaced by their inducible β-counterparts such as β1i (PMSB9 or LMP2), β2i (PMSB10 or MECL-1), and β5i (PMSB8 or LMP7), which form the immunoproteasome (Thibaudeau and Smith, 2019). Previous studies revealed that adverse stimuli activate the immunoproteasome, which is involved in the pathogenesis of cardiovascular diseases including cardiac hypertrophy, ischemia-reperfusion injury, and neointimal formation (Barringhaus and Matsumura, 2007; Li et al., 2015; Guo et al., 2016; Chen et al., 2019). Importantly, our recent studies indicate that Ang II infusion upregulates the activity and expression of immunoproteasome subunits (β2i and β5i) in the heart, atria, retina, and aorta (Li et al., 2015; Li et al., 2018; Wang et al., 2018; Li F. D. et al., 2019; Li J. et al., 2019; Wang et al., 2020). The depletion of β2i or β5i markedly attenuates Ang II-induced blood pressure, cardiac hypertrophy, and atrial fibrillation in mice (Li et al., 2015; Li et al., 2018; Li J. et al., 2019). Furthermore, we revealed that β5i is involved in the modulation of the infiltration of proinflammatory cells into abdominal aortic aneurysm and atherosclerotic lesion, as well as vascular remodeling in ApoE knockout mice (Li F. D. et al., 2019; Wang et al., 2020). It is worth noting that several nutritional factors, such as quercetin, δ-tocotrienol, and resveratrol, are potential proteasome inhibitors, which may represent a strategy for treating cardiovascular diseases (Qureshi et al., 2013; Chen et al., 2019). Here, we provided new evidence that GA administration significantly ameliorated the activity and expression of the immunoproteasome subunits β2i and β5i in Ang II-treated aortas (**Figure 3**). Therefore, these data suggest that GA serves as a novel inhibitor of the immunoproteasome and may reduce eNOS degradation in the aorta.

eNOS has been reported to play critical roles in the modulation of vasodilation, vascular inflammation, leucocyte adhesion, and vascular smooth muscle proliferation (Forstermann and Sessa, 2012). Knockout of eNOS impairs endothelium-dependent relaxation, elevates blood pressure, and induces abnormal vascular remodeling in mice (Forstermann and Sessa, 2012). Furthermore, drugs interfering with the RAAS, such as ACEIs and angiotensin receptor blockers, could improve eNOS dysfunction and vascular oxidative stress (Mancini et al., 1996; Wassmann et al., 2002). Early studies indicated that eNOS function is maintained by multiple mechanisms, including transcriptional and post-transcriptional modulation, post-translational modification, phosphorylation, and protein-protein interactions (Garcia and Sessa, 2019). Recently, increasing evidence has demonstrated that UPS-dependent proteolysis is responsible for the regulation of eNOS degradation (Stangl and Stangl, 2010). Proteasome inhibitors, including MG132, lactacystin, and MLN-273, increase eNOS expression in endothelial cells and in the arterial wall (Stangl et al., 2004; Herrmann et al., 2007; Thomas et al., 2007). In this study, we extended previous findings and revealed that GA markedly protected against the Ang II-induced degradation of eNOS by inhibiting the immunoproteasome subunits β2i and β5i, leading to an improvement of endothelial dysfunction (**Figures 2** **–** **4**). Accordingly, blocking eNOS activity with the inhibitor L-NAME significantly reversed these effects (**Figure 5**). Thus, our results indicate that GA attenuates hypertension and vascular remodeling by reducing the immunoproteasome-mediated degradation of eNOS.

In conclusion, this study unveiled a new role for GA in the regulation of hypertension and vascular dysfunction after Ang II stimulation. GA administration abolished the activity and expression of the immunoproteasome subunits β2i and β5i, which attenuated the degradation of eNOS. Thus, our findings suggest that GA is a new immunoproteasome inhibitor and may represent a promising therapeutic option for the treatment of hypertension and vascular remodeling. Further investigations are needed to explore the molecular mechanisms underlying the action of GA to modulate immunoproteasome expression.

## Data Availability Statement

The data that support the findings of this study are available from the corresponding author upon reasonable request.

## Ethics Statement

The animal study was reviewed and approved by the Animal Care and Use Committee of Capital Medical University (AEE1-2016-045) and conformed to the US National Institutes of Health Guide for the Care and Use of Laboratory Animals.

## Author Contributions

XY, Q-YZ, Y-LZ, and XH conducted the experiments. XY and Q-YZ analyzed the data. XY, S-BG, and H-HL designed the study. XY and H-HL wrote the manuscript and provided the funding for the study. XY and H-HL had primary responsibility for the final content. All authors contributed to the article and approved the submitted version.

## Funding

This work was supported by grants from the China Postdoctoral Science Foundation (2020M670384 to XY) and the National Natural Science Foundation of China (81703217 to XY, 81630009 and 81330003 to H-HL).

## Conflict of Interest

The authors declare that the research was conducted in the absence of any commercial or financial relationships that could be construed as a potential conflict of interest.

## Supplementary Material

Supplementary Figure S1 Effect of GA administration on mRNA expression levels of iNOS, AT1R, and AT2R. qPCR analyses ofthe mRNA expression of iNOS, AT1R, and AT2R in the aorta (n=6). GAPDH as the internal control. One-way ANOVA following Newman-Keuls multiple comparison test was utilized to evaluate the significance of difference between the means of groups.

Supplementary Figure S2 Effect of GA treatment on protein expression of p-eNOS1177, eNOS, β2i, and β5i in vitro. Representative immunoblotting analyses of the protein expression ofp-eNOS^1177^, eNOS, β2i, and β5iin confluent HUVEC streated with GA (10 μm) after 24 h of Ang II (100 nm) stimulation (left), and quantification of the relative protein levels (right, n=3). After the normality test (Shapiro-Wilk), the student t test was used to compare the significant difference between two groups in normal distribution, and the Mann-Whitney test was utilized for the data that were not normally distributed. **P* < 0.05 versus Ang II, #*P* < 0.05 versus Ang II + GA.
