# Supplementary figures and images for: Gallic Acid Attenuates Angiotensin II-Induced Hypertension and Vascular Dysfunction by Inhibiting the Degradation of Endothelial Nitric Oxide Synthase
Source: Front Pharmacol. 2020 Jul 22;11:1121. doi: 10.3389/fphar.2020.01121 (PMC7396711; doi:10.3389/fphar.2020.01121)

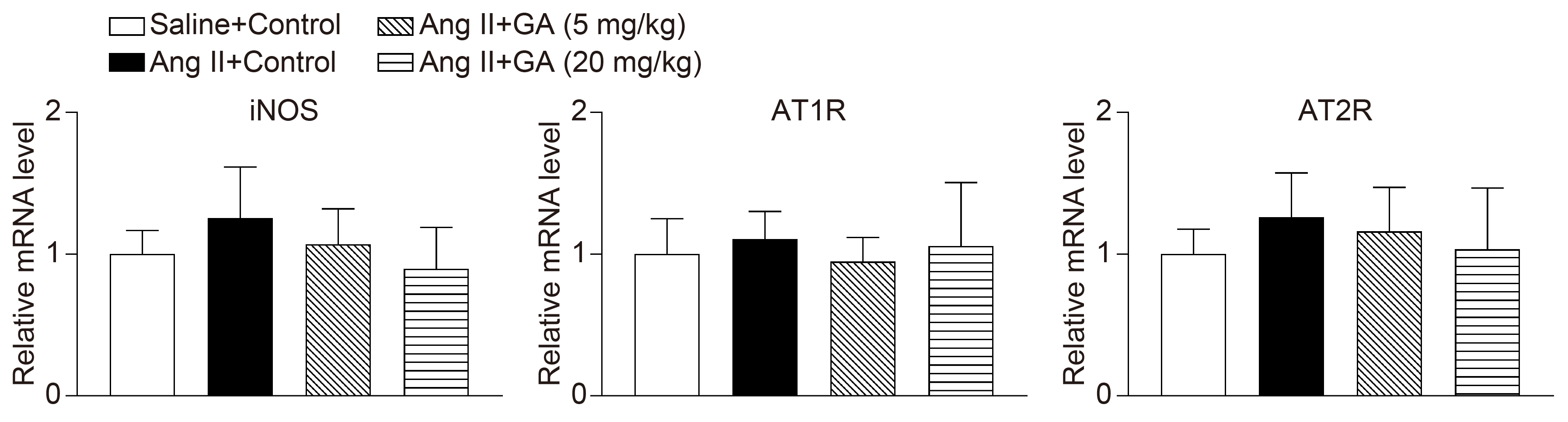

Supplement: Supplementary Figure S1 — Effect of GA administration on mRNA expression levels of iNOS, AT1R, and AT2R. qPCR analyses ofthe mRNA expression of iNOS, AT1R, and AT2R in the aorta (n=6). GAPDH as the internal control. One-way ANOVA following Newman-Keuls multiple comparison test was utilized to evaluate the significance of difference between the means of groups. [file Image_1.jpeg]

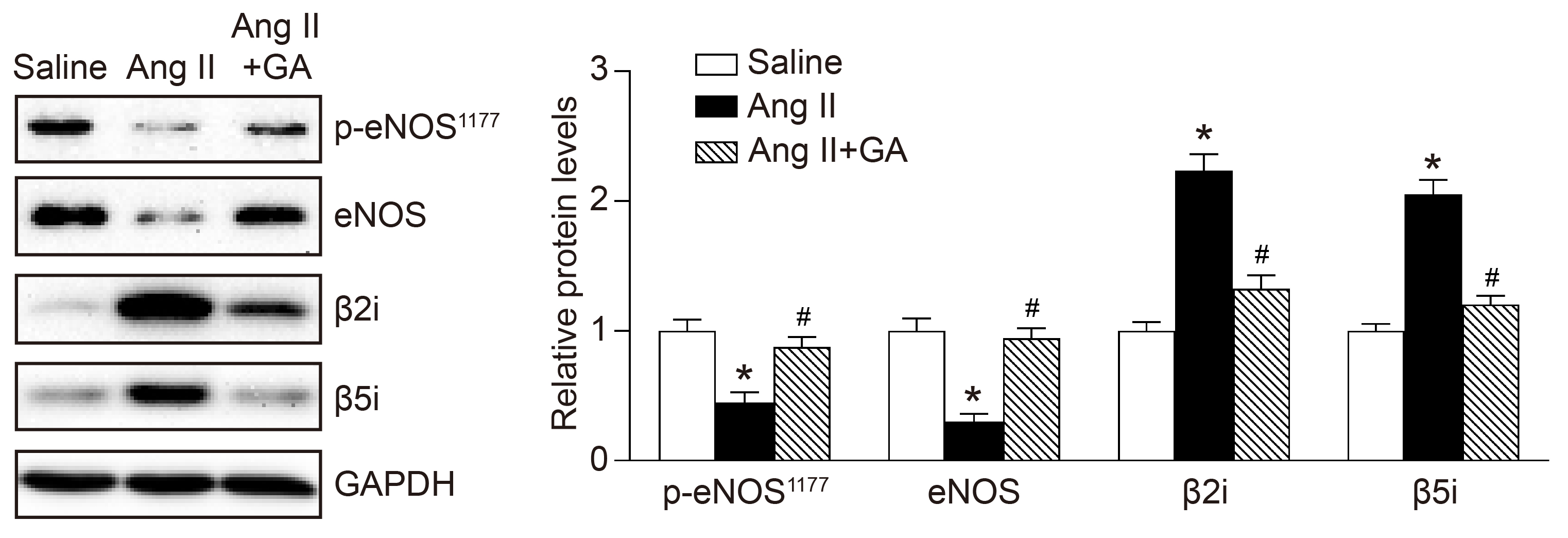

Supplement: Supplementary Figure S2 — Effect of GA treatment on protein expression of p-eNOS1177, eNOS, β2i, and β5i in vitro. Representative immunoblotting analyses of the protein expression ofp-eNOS1177, eNOS, β2i, and β5iin confluent HUVEC streated with GA (10 μm) after 24 h of Ang II (100 nm) stimulation (left), and quantification of the relative protein levels (right, n=3). After the normality test (Shapiro-Wilk), the student t test was used to compare the significant difference between two groups in normal distribution, and the Mann-Whitney test was utilized for the data that were not normally distributed. *P < 0.05 versus Ang II, #P < 0.05 versus Ang II + GA. [file Image_2.jpeg]
