# Supplementary material for: Gallic Acid Attenuates Angiotensin II-Induced Hypertension and Vascular Dysfunction by Inhibiting the Degradation of Endothelial Nitric Oxide Synthase
Source: Front Pharmacol. 2020 Jul 22;11:1121. doi: 10.3389/fphar.2020.01121 (PMC7396711; doi:10.3389/fphar.2020.01121)
Supplement: Supplementary file 3 [file Table_1.docx]

**Supplementary Table 1**. Primer sequences for RT-PCR analysis

IL-1β：interleukin-1β; IL-6：interleukin-6; TNF-α：tumor necrosis factor-α; MCP-1: monocyte chemoattractant protein-1; α-SMA, α-smooth muscle actin; NOX1: NADPH oxidase 1; NOX2: NADPH oxidase 2; NOX4, NADPH oxidase 4; p22^phox^, cytochrome b-245, alpha polypeptide; eNOS, endothelial nitric oxide synthase; iNOS, Inducible nitric oxide synthase; AT1R, Ang II type 1 receptor; AT2R, Ang II type 2 receptor; GAPDH, glyceraldehyde 3-phosphate dehydrogenase.

| Gene | Forward primer (5’-3’) | Reverse primer (5’-3’) |
| --- | --- | --- |
| IL-1β | CTTCCCCAGGGCATGTTAAG | ACCCTGAGCGACCTGTCTTG |
| IL-6 | TTCCATCCAGTTGCCTTCTTG | TTGGGAGTGGTATCCTCTGTGA |
| TNF-α | ATGGCCTCCCTCTCATCAGT | CTTGGTGGTTTGCTACGACG |
| MCP-1 | AGCTGTAGTTTTTGTCACCAAGC | GTGCTGAAGACCTTAGGGCA |
| α-SMA | TCCTGACGCTGAAGTATCCGATA | GGCCACACGAAGCTCGTTAT |
| Collagen I | GAGTACTGGATCGACCCTAACCA | GACGGCTGAGTAGGGAACACA |
| Collagen III | TCCCCTGGAATCTGTGAATC | TGAGTCGAATTGGGGAGAAT |
| NOX1 | CCCATCCAGTCTCCAAACATGAC | ACCAAAGCTACAGTGGCAATCAC |
| NOX2 | CTTCTTGGGTCAGCACTGGC | GCAGCAAGATCAGCATGCAG |
| NOX4 | CTTGGTGAATGCCCTCAACT | TTCTGGGATCCTCATTCTGG |
| p22^phox^ | CTCCTCTTCACCCTCACTCG | GTGGACTCCCATTGAGCCTA |
| eNOS | TCAGCCATCACAGTGTTCCC | ATAGCCCGCATAGCGTATCAG |
| β1 | CCAATCGAGTGACTGACAAGCT | GGACTAGTGGAGGCTCGTTCA |
| β2 | AGGCCAGATATGGAGGAGGAA | GGGCACTGAGAATGGACGAA |
| β5 | TGCTCGCTAACATGGTGTATCAGTA | AGCCAGAGCCCACTGAGAAG |
| β1i | CTGGAGCTACACGGGTTGGA | ATATACCTGTCCCCCCTCACATT |
| β2i | CAGCCGTCTGCCCTTTACTG | AGAGCCCAGGTCACTCAGGAT |
| β5i | CTTGGCACCATGTCTGGTTGT | CCGGTACTGCAGCATCATGT |
| iNOS | GTTCTCAGCCCAACAATACAAGA | GTGGACGGGTCGATGTCAC |
| AT1R | AACAGCTTGGTGGTGATCGTC | CATAGCGGTATAGACAGCCCA |
| AT2R | AACTGGCACCAATGAGTCCG | CCAAAAGGAGTAAGTCAGCCAAG |
| GAPDH | GGTTGTCTCCTGCGACTTCA | GGTGGTCCAGGGTTTCTTACTC |
